# Adipose-derived stem cells promote the repair of chemotherapy-induced premature ovarian failure by inhibiting granulosa cells apoptosis and senescence

**DOI:** 10.1186/s13287-023-03297-5

**Published:** 2023-04-11

**Authors:** Guihai Ai, Meng Meng, Jing Guo, Caixia Li, Jihui Zhu, Li Liu, Biting Liu, Wenhan Yang, Xiaowen Shao, Zhongping Cheng, Lian Wang

**Affiliations:** 1 Department of Gynecology and Obstetrics, Shanghai Tenth People’s Hospital, Tongji University School of Medicine, Shanghai, 200072 China; 2 Gynecologic Minimally Invasive Surgery Research Center, Shanghai Tenth People’s Hospital, Tongji University School of Medicine, Shanghai, 200072 China; 3 Tongji University School of Medicine, Shanghai, 200092 China; 4 Department of Gynecology and Obstetrics, Shanghai First Maternity and Infant Hospital, Tongji University School of Medicine, Shanghai, 200040 China

**Keywords:** Premature ovarian failure, Adipose-derived stem cells, Microenvironment, Cellular senescence, PI3K/Akt/mTOR axis

## Abstract

**Background:**

Chemotherapeutic drugs, particularly alkylating cytotoxics such as cyclophosphamide (CTX), play an important role to induce premature ovarian failure (POF). Hormone replacement therapy (HRT) is a widely used treatment to improve hormone secretion. However, the long-term HRT increases the risk of breast cancer and cardiovascular disease are attracting concerns. Therefore, there is an urgent need to develop a safe and effective treatment for POF.

**Method:**

Adipose-derived stem cells (ADSCs) were isolated and identified from human adipose tissue. For POF modeling, CTX were intraperitoneal injected into CTX-acute group, CTX-chronic group, CTX-acute + ADSCs group and CTX-chronic + ADSCs group rats; For transplantation, ADSCs were transplanted into POF rats through tail-vein. The control group rats were injected with PBS. The effects of POF modeling and transplantation were determined by estrous cycle analysis, histopathological analysis, immunohistochemical staining and apoptosis-related marker. To evaluate the effects of ADSC on granulosa cells in vitro, CTX-induced senescent KGN cells were co-cultured with ADSCs, and senescent-related marker expression was investigated by immunofluorescent staining.

**Results:**

In vivo studies revealed that ADSCs transplantation reduced the apoptosis of ovarian granulosa cells and secretion of follicle-stimulating hormone. The number of total follicles, primordial follicles, primary follicles, and mature follicles and secretion of anti-Müllerian hormone and estradiol (E2) were also increased by ADSCs. The estrous cycle was also improved by ADSC transplantation. Histopathological analysis showed that CTX-damaged ovarian microenvironment was improved by ADSCs. Furthermore, TUNEL staining indicated that apoptosis of granulosa cells was decreased by ADSCs. In vitro assay also demonstrated that ADSC markedly attenuated CTX-induced senescence and apoptosis of granulosa cell. Mechanistically, both in vivo and in vitro experiments proved that ADSC transplantation suppressed activation of the PI3K/Akt/mTOR axis.

**Conclusion:**

Our experiment demonstrated that a single injection of high-dose CTX was a less damaging chemotherapeutic strategy than continuous injection of low-dose CTX, and tail-vein injection of ADSCs was a potential approach to promote the restoration of CTX-induced POF.

## Introduction

Premature ovarian failure (POF) is a common gynecological disease and the leading cause of infertility in women under the age of 40 [1, 2]. The pathogenesis of POF is a complex and multifactorial process. Studies have indicated that various factors including genetic [3, 4], autoimmune [5, 6], and iatrogenic factors [2, 7] and environmental stress [8] are associated in the dysregulation of ovarian structure and function, and chemo and radiotherapy have gradually become the major reasons leading to POF [9, 10]. Statistical data confirmed that as the overall survival of patients with cancer was significantly prolonged, the incidence of POF dramatically increased to 2% in women under the age of 40 [11]. Damaged ovarian structures and dysfunctional hormone secretion lead to irregular menstruation, amenorrhea [1] and infertility [12] in patients with POF. Patients often suffer from urogenital symptoms such as vaginal dryness, sexual intercourse pain, and dysuria, as well as neuropsychiatric symptoms including irritability, depression, anxiety and memory loss, which seriously affect quality of life [13]. Currently, there is still a lack of effective treatments to protect and restore ovarian secretory function. Existing therapies targeting to specific clinical symptoms have been developed, including hormone replacement therapy (HRT) [14], induced ovulation therapy [15, 16] and ovarian tissue or oocyte transplantation [17]. However, these treatments barely improve endocrine and reproductive functions of the ovary and are associated with serious adverse effects, such as long-term injection of contraceptive steroid hormones, which significantly increase the risks of breast cancer [18, 19], cardiovascular disease [20] and stroke [21]. Therefore, there is an urgent need to develop a safe and effective treatment for POF.

Adipose-derived stem cells (ADSCs) have been widely used to treat diabetic foot [22, 23], rheumatoid arthritis [24, 25], knee osteoarthritis [26], Crohn's disease [27, 28] and cirrhosis [29] due to their abundance, convenient access and low immunogenicity [30]. An increasing number of studies have confirmed that ADSCs have great potential to restore the structure and function of damaged tissues and provide new treatment methods for various refractory diseases, such as POF [31]. Takehara et al. [32] confirmed that ADSCs promoted angiogenesis in the cyclophosphamide (CTX)-injured ovarian tissue. The researchers proved that ADSC transplantation significantly increased the secretion of vascular endothelial growth factor (VEGF), insulin-like growth factor-1 (IGF-1) and hepatocyte growth factor (HGF), indicating that ADSCs play an important role in restoring the number of ovarian follicles and corpus luteum. Currently, studies have confirmed the benefit of ADSCs in the treatment of POF; however, the mechanism remains to be elucidated. Akt/mTOR is one of the most widely distributed activators of damage-induced physiological processes. Akt/mTOR activation is closely involved in cell proliferation [33], migration [34], differentiation [35], apoptosis [36] and autophagy [37]. Inhibitory factors, including ROS, starvation, hypoxia, inflammation, and other stress conditions, reduce the phosphorylation of PI3K/Akt and the downstream effector mTOR, causing the dysregulation of PI3K/Akt/mTOR-induced cell death and abnormal tissue structure and function [38]. During the development of ovarian follicles, Akt hyperactivation activates the downstream effector mTOR in follicles and inhibits autophagy in granulosa cells in ovarian tissue, which might be involved in POF. Therefore, we will examine whether the treatment of POF by ADSC transplantation occurs through the regulation of the PI3K/Akt/mTOR pathway.

In this study, we established CTX-induced acute and chronic POF rat models, which enabled us to examine the protective and reparative effects of ADSCs on ovarian tissue. To evaluate the degree of damage to follicles and granulosa cells induced by CTX, we established a rat POF in vivo model and an in vitro cellular senescence model and assessed apoptosis and senescence in granulosa cells, which might be an important trigger of estrogen deficiency and infertility. Furthermore, we examined the mechanism by which ADSC transplantation ameliorates POF, suggesting a theoretical foundation for the clinical application of ADSCs.

## Materials and methods

### Animals

Eight-week-old female Sprague Dawley rats (SD rats) were purchased from Shanghai SLAC Laboratory Animal Co. Ltd and kept for 7 days for adaption and to protect against any health problems. Throughout the experiments, the rats were maintained at 22 °C with a 12-h/12-h light/dark cycle and free access to a standard pellet diet and water. All animal experiments followed the animal care requirements of the Ethics Committee of the Tenth’s Hospital of Tongji University.

### Culture of KGN granulosa cell line

KGN cell line was purchased from MeiSenCTCC company (human KGN cell line, CTCC-003-0105) and maintained in DMEM/F12 medium supplemented with 10% fetal bovine serum (FBS, Gibco, 10,099–141). KGN cell line were cultured at 37 °C in an incubator with 5% CO_2_.

### Establishment of the acute and chronic POF rat models

The acute POF and the chronic POF model were established, respectively. To establish the acute POF rat model, female SD rats (*n* = 12) weighting between 200 and 250 g with regular estrous cycles were intraperitoneal injected with 140 mg/kg CTX (Selleck Chemicals, S1217). To establish the chronic POF rat model, female SD rats (*n* = 12) weighting between 200 and 250 g with regular estrous cycles were intraperitoneal and continuously injected with 20 mg/kg/day CTX for 7 days. Control group rats (*n* = 12) were administered the same volume of PBS via intraperitoneal injection.

### Isolation and identification of human ADSC

The isolation and identification of human ADSC were performed according to a previously reported protocol [39]. In brief, adipose tissue was washed with PBS and minced into pieces of 1 mm^3^ with scissors, then the tissue was digested with 0.1% type I collagenase (Gibco, 17,100,017) for 60 min in 37 °C water baths. Undigested debris were removed by using 100 μm cell strainer. Then, after centrifugation for 5 min at 2000 rpm, the cell pellet was resuspended and plated in T25 flask and cultured in 5 mL of DMEM/F12 medium supplemented with 10% FBS and passaged until 90% confluence. The medium was changed every other day. Flow cytometry and tri-differentiation assays were performed to identify the cell surface markers (Human MSC Analysis Kit, BD Bioscience, 562,245) and multipotency of human ADSCs as previously reported. Fifth-passage human ADSCs were used for all transplantation experiments.

### ADSC transplantation

One week after POF models were established, ADSCs were transplanted into acute and chronic POF rats. Briefly, the rats were randomly divided into five groups: Control (*n* = 15), CTX-acute group (*n* = 15), CTX-acute + ADSC group (*n* = 15), CTX-chronic group (*n* = 15) and CTX-chronic + ADSC group (*n* = 15). For transplantation of ADSCs, 1 × 10^6^ fifth-passage human ADSCs resuspended in 100 μL of PBS were transplanted into acute and chronic POF rats through tail-vein injections. PBS (100 μL) was injected into rats in the control group.

### Estrous cycle analysis

To evaluate the estrous cycles of rats, vaginal smears were performed daily before 8 a.m. for a consecutive 8-day cycle. Briefly, 10 μL of distilled water was used to flush the vagina, and the fluid was then collected and plated on a glass slide. The stage of the estrous cycle was determined with a light microscope. The proportion of leukocytes, nucleated epithelial cells, and cornified squamous epithelial cells was used to distinguish the four phases of the estrous cycles [40, 41]. A proestrus smear consists of a predominance of nucleated epithelial cells. An estrous smear primarily consists of a nucleated cornified cells. A metestrus smear consists of the same proportion among leukocytes, cornified, and nucleated epithelial cells. And a diestrus smear primarily consists of a predominance of leukocytes.

### HE staining to analyze ovarian morphology and follicle counting

To assess the number of follicles and morphological changes in POF rats ovaries, rats were sacrificed after 1, 7, 14 and 28 days of injection of CTX, ovaries were collected used for histological staining. To evaluate the therapeutic efficacy of ADSCs on the number and morphology of POF rat’s ovary, rats were sacrificed after 1, 7, 14 and 28 days of ADSC transplantation, ovaries were collected used for histological staining. Briefly, Ovaries were fixed with 10% neutral formalin for 24 h, and then the ovaries were gradient-dehydrated with 20% and 30% sucrose until the tissue completely sank to the bottom. Following paraffin embedding, the ovaries were serially sectioned at a thickness of 5 μm, and then mounted on glass slides. The sections were stained with hematoxylin and eosin (H&E, Solarbio, G1120) solution for follicles counting and morphological observation. The continuous section of total follicles, primordial follicles, primary follicles and mature follicles (secondary and atresia follicles) were counted at intervals of 50 μm under Olympus BX53 microscope.

### Immunohistochemistry and immunofluorescent staining

The expression of apoptosis-, proliferation-, organ damage repair-related cytokines; and AKT-mTOR signaling factors were evaluated by immunohistochemistry. Paraffin-embedded sections were dewaxed, rehydrated, and blocked with 5% bovine serum albumin for 30 min at RT. Then, the sections were incubated with the following primary antibodies at 4 °C overnight: anti-rat Ki67 (1:100, Thermo Fisher Scientific, MA5-14,520), anti-rat VEGF (1:100, Thermo Fisher Scientific, MA5-13,182), anti-rat HGF (1:100, Thermo Fisher Scientific, PA5-115,354), anti-rat IGF-1 (1:100, Thermo Fisher Scientific, MA1-088), anti-rat AKT (1:100, Thermo Fisher Scientific, MA5-14,916), anti-rat mTOR (1:100, Thermo Fisher Scientific, PA1-518), anti-rat FOXO3a (1:100, Thermo Fisher Scientific, MA5-14,932) and anti-rat RPS6 (1:100, Thermo Fisher Scientific, 710,405). After that the anti-rabbit IgG secondary antibody (1:500, Thermo Fisher Scientific, A16110) was added and incubated with the washed sections for 1 h at 37 °C and visualized using diaminobenzidine substrate (Thermo Fisher Scientific, 34,002). The immunostained sections were observed and imaged under Olympus BX53 microscope, and then the area of interests of IHC staining were quantified using ImageJ 1.52a software IHC profiler. High positive (3+), Positive (2+), Low Positive (1+) and Negative (0).

The granulosa cell line KGN was fixed in 4% PFA for 15 min at RT. The fixed cells were incubated with 0.3% Triton X-100 in PBS for 15 min. Then, the cells were incubated with 3% BSA blocking buffer for 1 h at RT. The cells were incubated with following primary antibodies at 4 °C overnight: anti-rat Ki67 (1:1000), anti-rat p21 (1:1000, Thermo Fisher Scientific, PA1-30,399), anti-rat AKT (1:1000), anti-rat mTOR (1:1000), anti-rat FOXO3a (1:1000) and anti-rat RPS6 (1:1000). After that the cells were incubated with anti-rabbit Cy3 antibody (1:1000, Thermo Fisher Scientific, A10520) for 2 h at RT. Nuclei were counterstained with DAPI. All images were observed and analyzed by Nikon A1R microscope with NIS-Elements Viewer 4.5 software.

### Senescence-associated *β*-Galactosidase staining

SA-*β*-Gal activity was measured in the KGN granulosa cell line using Senescence-associated *β*-Galactosidase Staining kit (Beyotime, C0602) according to the manufacturer’s instructions. Briefly, KGN cells were seeded in 24-well plate and treated with CTX or CTX + ADSC. At the end of the experiment, KGN cells were washed with PBS and fixed with 4% PFA. Then, KGN cells were incubated with *β*-Gal substrate at 37 °C overnight. After being washed with PBS, positive senescent cells were imaged with a Nikon A1R microscope and counted with Image J 1.52a software.

### MitoTracker red CMXRos/annexin V FITC staining

To assess the apoptosis of KGN cells, the MitoTracker red CMXRos/Annexin V FITC staining kit (Beyotime, C1035) was used according to the manufacturer’s instructions. Briefly, at the end of the experiments, KGN cells were harvested and stained with MitoTracker CMXRos and Annexin V FITC dye solution. After being incubated for 30 min at RT, the stained cell was imaged under the Nikon A1R microscope with NIS-Elements Viewer 4.5 software.

### Enzyme-linked immunosorbent assay

In this study, rats were treated under indicated conditions. The blood samples of rats were collected under indicated timepoints, and then centrifuged to clear the cell debris. AMH (Abbkine, KTE100996), FSH (Abbkine, KTE100733) and E2 (Abbkine, KTE100947) levels in serum samples were determined by the ELISA assay. The serum was diluted by 1:100 with ELISA dilution buffer. All experiments were performed according to the manufacturer’s instructions.

### Statistical analysis

All data were collected at the end of experiment and analyzed using GraphPad Prism 9.0.0 software. The results were exhibited as the mean ± SD. One-Way ANOVA test was used to assess significance among different groups. *p* value < 0.05 was significant in statistical analysis results.

## Results

### Isolation and identification of ADSCs

**Fig. 1:**
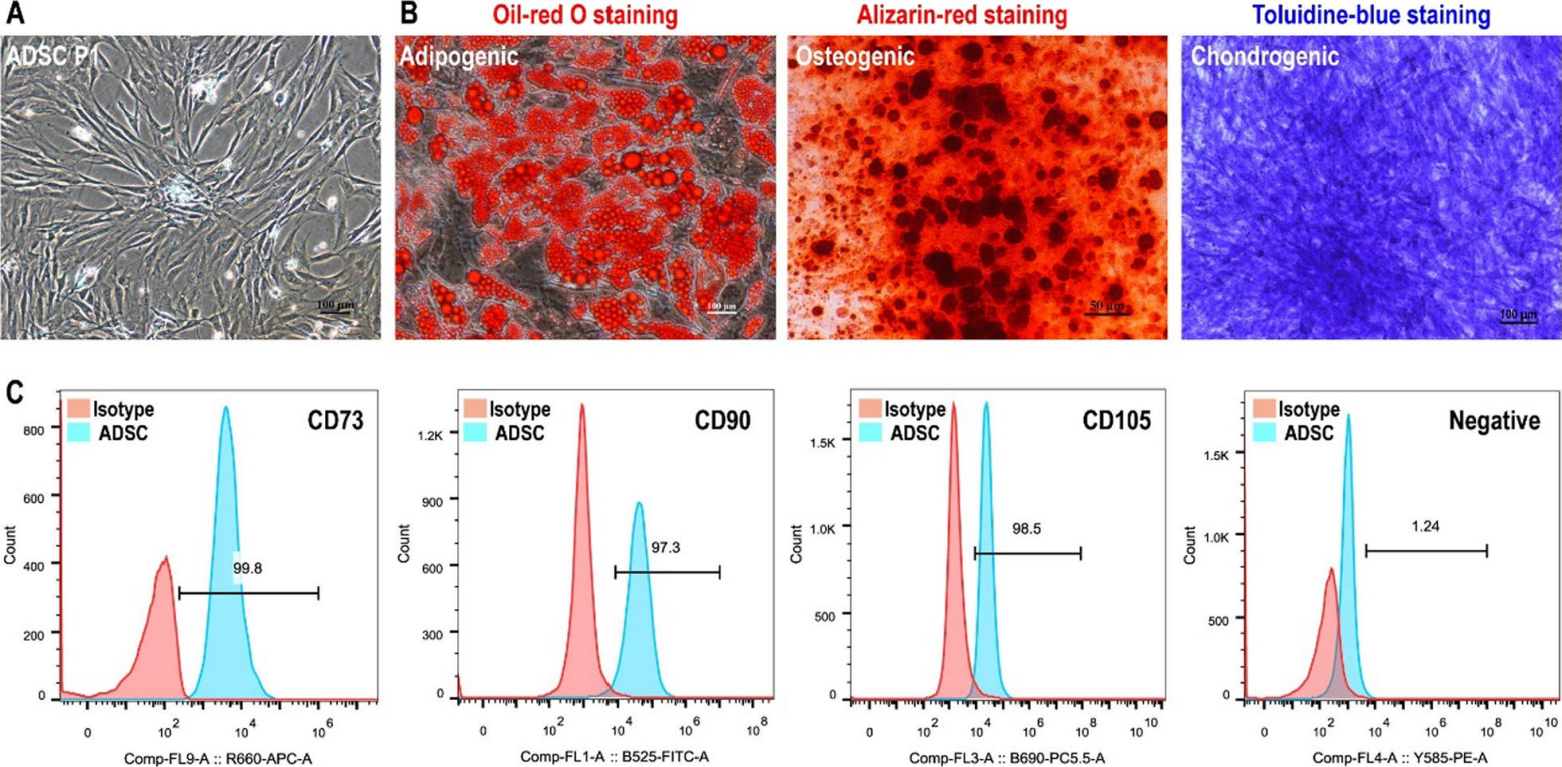
Isolation and identification of human ADSCs. **A** The isolated ADSCs exhibited typical fibroblast-like morphology. Bar: 100 μm. **B** Induction of ADSCs into adipocytes, osteoblast, and chondrocytes with adipogenic, osteogenic and chondrogenic medium, respectively. Bar:100 μm. **C** ADSCs showed strong positive (more than 95%) expression of CD73, CD90 and CD105, and negative expression (less than 5%) of negative cocktail including CD11b, CD19, CD45, and HLA-DR

Human ADSCs were isolated from adipose tissue according to previous published paper [39]. Isolated ADSCs were cultured in DF12 containing 10% FBS and 20 ng/mL bFGF in 6-well plates. ADSCs exhibited typical spindle fibroblast-like morphology (Fig. 1A). To identify the multipotency of ADSCs, adipogenic, osteogenic and chondrogenic differentiation assays were performed. The results of oil-red O staining, alizarin red staining, and toluidine blue staining proved that ADSCs displayed multipotency to differentiate into adipocytes, osteocytes, and chondrocytes (Fig. 1B). Flow cytometry was performed to verify cell surface markers on ADSCs. The results demonstrated that ADSCs showed strong positive (more than 95%) expression of CD73, CD90 and CD105, and negative expression (less than 5%) of CD11b, CD19, CD45 and HLA-DR (Fig. 1C).

### Long-term injection of low-dose CTX induces more damage to ovarian tissue

**Fig. 2:**
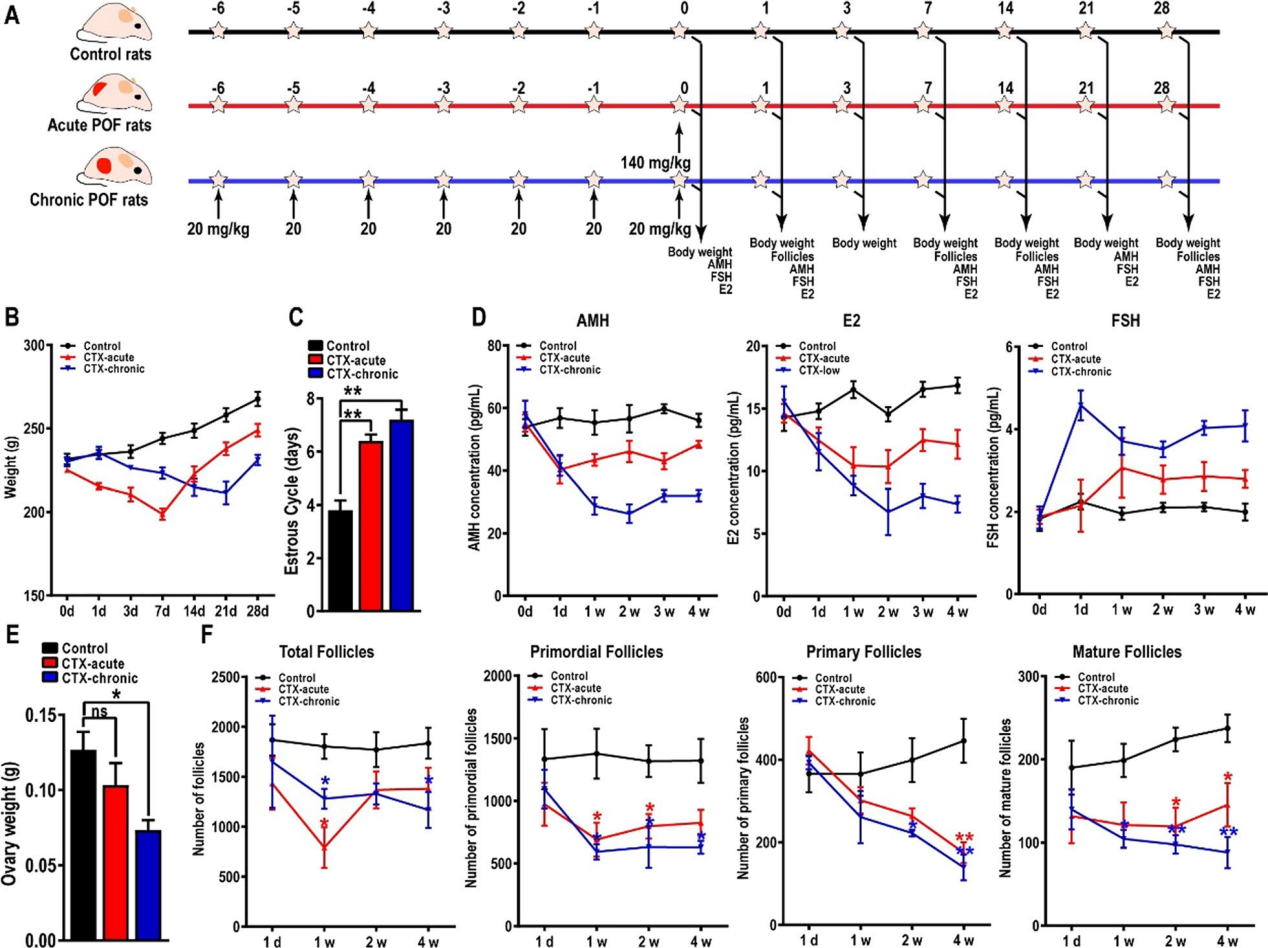
Establishment of the acute POF rat model and the chronic POF rat model. (**A**) The process of establishment of the acute POF rat model and the chronic POF rat model. To confirm model was established, body weight (**B**), estrous cycle (**C**), hormone secretion (**D**), ovary weight (**E**) and the number of follicles (**F**) were evaluated

Chemotherapeutic drugs including CTX typically induces impairment of structure and endocrine function in ovarian follicles and granulosa cells. The extent of ovarian damage depends mainly on the toxicity and dose of CTX. To better understand the response of ovarian tissue to different doses of CTX, we established acute POF model by using a single injection of high-dose CTX (140 mg/kg) and chronic POF model by injections of low-dose CTX (20 mg/kg) for 7 continuous days (Fig. 2A). Our data showed a dramatic decrease in body weight of the acute POF group rats (19.51 ± 2.40%, 7 days), however, prolonged slow weight loss in the chronic POF group rats (19.05 ± 4.55%, 21 days). Body weight of the acute POF group recovered faster than in chronic POF group (Fig. 2B). We next assessed the estrous cycle in acute and chronic POF rats. The results demonstrated that the estrous cycle was significantly extended in acute and chronic POF rats, or was even undetectable (Fig. 2C). To evaluate the impairment of CTX on the hormone secretion in acute and chronic POF rats, the serum levels of AMH, E2 and FSH were examined by using an ELISA kit according to the manufacturer’s instructions. As expected, AMH and E2 levels were significantly reduced, and FSH levels were increased, which consistent with the symptoms of POF. More importantly, compared to the acute POF rats, chronic POF rats secreted significantly less AMH and E2, and secreted more FSH (Fig. 2D). Consistent with the body weight, continuous injection of low-dose CTX induced more ovary weight loss than a single injection of high-dose CTX (Fig. 2E). Then, to directly investigate the damage to CTX on ovarian structure, HE staining was performed to examine the numbers of total follicles, primordial follicles, primary follicles, and mature follicles. Based on the results of follicle count, the damage to ovarian tissue caused by CTX gradually appeared after one week. As shown in Fig. 2, one week after POF modeling, the total follicles of acute POF (791 ± 353) and chronic POF (1280 ± 173) mice were significantly decreased compared to normal mice (1804 ± 216). After 4 weeks of modeling, the number of total follicles in acute POF (1380 ± 366) and chronic POF (1168 ± 312) mice partially recovered, but still less than control mice (1835 ± 270). Then the number of primordial follicles, primary follicles and mature follicles in rats were further counted. As shown in Fig. 2, the number of primordial follicles in CTX-chronic POF rats (825 ± 179) and CTX-acute POF rats (630 ± 89) were significantly decreased in control mice (1319 ± 304, *, *p* < 0.05). Consistently, the number of primary follicles was also decreased in CTX-chronic POF rats (139 ± 54) and CTX-acute POF rats (174 ± 43) compared to control mice (445 ± 91). Lastly, the number of mature follicles was also decreased in CTX-chronic POF rats (88 ± 32) and CTX-acute POF rats (145 ± 45) compared to control mice (237 ± 28). These results collectively confirmed the successful establishment of the POF rat model and suggested that long-term continuous injection of low-dose CTX induced more damage to ovarian structure and function (Fig. 2F).

### Hormone secretion and ovarian structure were restored after ADSC transplantation

**Fig. 3:**
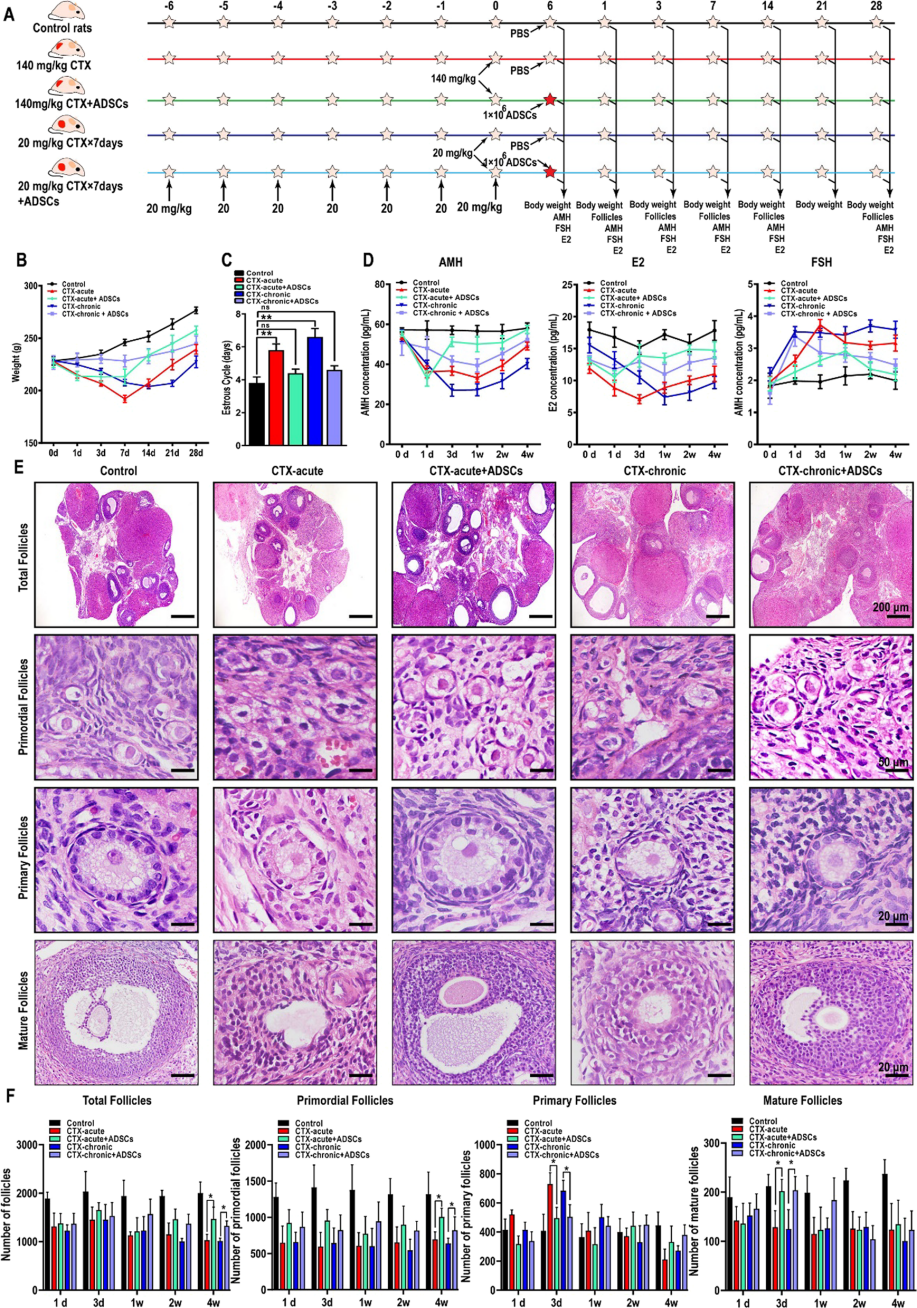
Hormone secretion and ovarian structure restored after ADSC transplantation. **A** The process of ADSC transplantation. **B** The body weight of acute and chronic POF rats were obviously increased after ADSC transplantation. **C** ADSC transplantation restored the disrupted estrous cycle. **D** ELISA assay demonstrated that ADSC transplantation enhanced the level of AMH and E2 and decreased the level of FSH. **E**–**F** The structure and number of follicles were restored in ovarian tissue of ADSCs transplanted rats. Scale bar: 200, 50, 20, 20 μm

We then investigated the therapeutic potential of ADSCs in promoting of ovarian tissue restoration. Sprague Dawley rats were divided equally into five groups, the control group, acute POF group, acute POF + ADSC group, chronic POF group, and chronic POF + ADSC group. 1 × 10^6^ in 100 μL ADSCs were transplanted into acute and chronic POF rats by tail-vein injection, and the control group of rats was injected with PBS as a control (Fig. 3A). First, the body weight of rats was measured after ADSC transplantation. The results indicated that compared to those of untreated POF rats, the body weights of ADSC-transplanted POF rats were significantly increased (Fig. 3B). Subsequently, the vaginal smear was performed to assess the estrous cycle. The results indicated that the disturbed estrous cycles of rats with acute or chronic POF were also restored to almost normal levels due to ADSC transplantation (Fig. 3C). To evaluate the repairment effect of ADSCs on ovarian hormone secretion, we measured the serum level of AMH, E2, and FSH in rat serum samples. After ADSC transplantation, the serum level of AMH and E2 were significantly enhanced, indicated that the fertility and hormone secretion had markedly improved. Notably, as a POF marker, FSH secretion was increased in acute and chronic POF rats and was significantly reduced by ADSC transplantation. These results revealed that the therapeutic potential of ADSCs in treating POF (Fig. 3D). We next examined whether ADSC transplantation improves the ovarian tissue recovery in POF rats. Histopathological analysis showed that the ovarian cortex was severely damaged in POF rats. Interestingly, these changes were coupled with hypertrophy and atresia of follicles. Importantly, these disorders were alleviated by ADSC transplantation, the numbers of primordial follicles, primary follicles, and mature follicles were increased (Fig. 3E). In contrast, we also found that primary follicles in acute and chronic POF rats rebounded on Day 3 after modeling (Fig. 3E). The potential mechanism underlying this process might be the activation and development of primordial follicles induced by CTX in a short time, which exhausts fertility preservation. Furthermore, in acute and chronic POF rats, the morphological results demonstrated that a number of follicles survived in an unhealthy state with a disordered arrangement of oocytes and granulosa cells. In ADSC-treated ovarian tissue, although the quantity of follicles was less than that in control group rats, granulosa cells surrounded the oocytes neatly and regularly in the surviving follicles, indicating a healthy follicle structure similar to that of the control group (Fig. 3E). To further evaluate the repairment role of ADSC transplantation, the number of total follicles, the primordial follicles, primary follicles and mature follicles were counted. As shown in Fig. 3, the number of total follicles was recovered in CTX-acute + ADSC rats (1464 ± 209) and CTX-chronic + ADSC rats (1371 ± 193) than in CTX-acute POF rats (1151 ± 234) and CTX-chronic POF rats (1005 ± 60). Consistently, the primordial follicles, primary follicles and mature follicles were also significantly increased in ADSC transplantation rats (Fig. 3F).

### Angiogenesis and wound healing-associated cytokines increased after ADSC transplantation

**Fig. 4:**
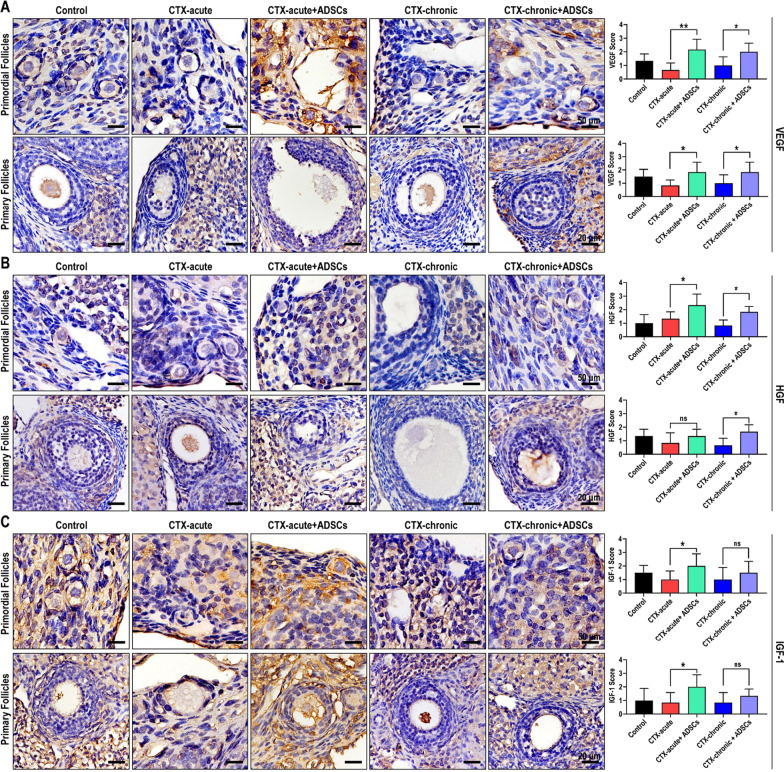
Angiogenesis and wound healing-associated cytokines increased after ADSC transplantation. ADSC transplantation promotes the expression of VEGF (**A**), HGF (**B**), and IGF-1 (**C**) in ovarian tissue

Cytokines including VEGF, HGF and IGF-1 are involved in regulating cell survival, proliferation, differentiation, and migration, and therefore play important roles in promoting the repair of damaged ovarian tissues. To investigate the effect of ADSCs on these cytokines, we assessed the levels of VEGF, HGF and IGF-1 in ovarian tissues by immunohistochemical staining. Our results suggested that the expression of VEGF in ovarian granulosa cells was decreased in acute and chronic POF rats, hindering the repair of damaged ovarian tissues. After treatment with ADSCs, ovarian function was promoted, perhaps partly due to ADSCs significantly increasing VEGF levels in ovarian granulosa cells (Fig. 4A). Immunohistochemical staining of HGF in acute and chronic POF rats obtain similar results. The expression of HGF was decreased in ovarian granulosa cells in acute and chronic POF rats and was promoted by ADSC transplantation. HGF is a cytokine that promotes the growth and differentiation of granulosa cells, providing another mechanistic explanation by which ADSCs repair damage (Fig. 4B). IGF-1 is widely distributed in ovarian tissue and has an antiapoptotic effect. In rats with acute and chronic POF, there were strong positive signals of apoptosis of follicles and granulosa cells in ovarian tissue, along with a significant reduction in IGF-1 expression in granulosa cells. In contrast, immunohistochemical analysis of IGF-1 confirmed that treatment with ADSCs upregulated the expression of IGF-1 in granulosa cells, thereby increasing the antiapoptotic capacity of ovarian tissues. (Fig. 4C). We conclude that ADSC transplantation increases the levels of various cytokines in granulosa cells and supports a microenvironment that is conducive to apoptosis inhibition, regeneration, and the repair of damaged ovarian tissue.

### Apoptosis and senescence of granulosa cell decreased after ADSC transplantation

**Fig. 5:**
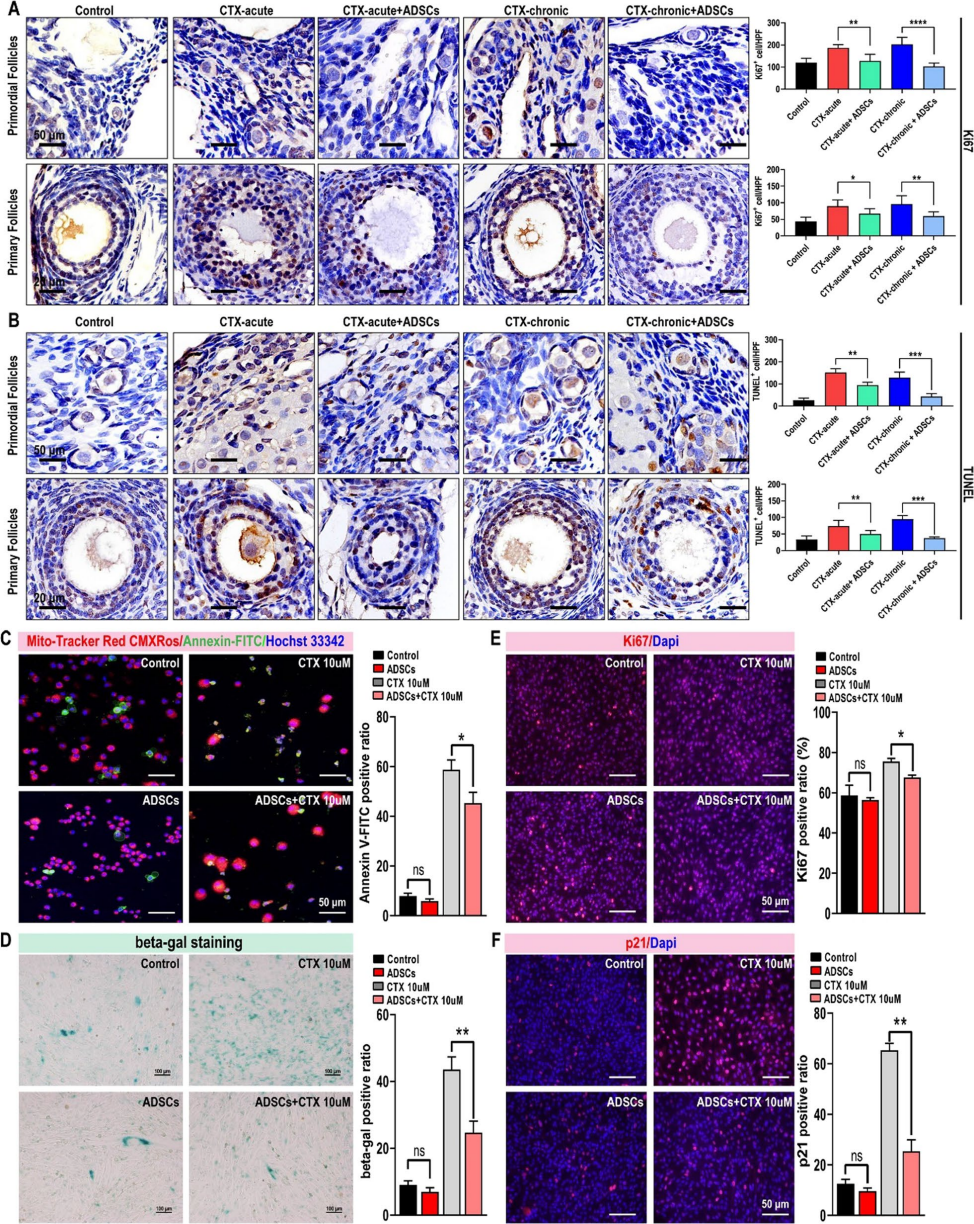
Apoptosis and senescence of granulosa cell decreased after ADSC transplantation. The IHC staining results revealed that the proliferation (**A**) and apoptosis (**B**) were increased in rats with POF and decreased in rats treated with ADSCs. Live and dead cell staining (**C**) and SA-β-gal staining (**D**) showed that CTX increased the cell death and senescence of granulosa cells, and co-cultured with ADSCs reduced the death and senescence. **E** Ki67 staining of CTX-treated KGN cell and ADSCs co-cultured cells. **F** p21 staining of CTX-treated KGN cells and ADSCs co-cultured cells

The accumulated damage in ovarian granulosa cells and follicular atresia mediated by CTX cytotoxicity might, in part, explain the reductions in ovarian endocrine function and fertility. Apoptosis and cellular senescence are different biological effects induced by chemotherapeutic drugs. We suggest that CTX induces apoptosis and senescence in follicular and ovarian granulosa cells, resulting in functional impairment. To verify protective effect of ADSCs on granulosa cells, Ki67 and TUNEL staining were performed to examine cell proliferation and apoptosis in rat ovarian tissue. The results showed that CTX increased Ki67-positive granulosa cells in the primordial and primary follicles of rats with acute and chronic POF, and proliferative cells were more sensitive to CTX, resulting in increased apoptosis and severe damage to ovarian tissue. However, ADSC transplantation inhibited the proliferation of ovarian primordial and primary follicles, thereby rendering granulosa cells insensitive to CTX and reducing the number of apoptotic cells (Fig. 5A–B). Therefore, our results showed that apoptosis and senescence in granulosa cells induced by CTX was the major reason for abnormal follicular structure and irregular endocrine function. To directly examine the effect of CTX on granulosa cells and exclude interference from complicated factors in vivo, we established an in vitro granulosa cell line model to mimic CTX-induced apoptosis and cellular senescence. MitoTracker Red/Annexin FITC staining (Fig. 5C) and *β*-gal staining (Fig. 5D) proved that compared to control and ADSC co-cultured granulosa cells, CTX significantly increased apoptosis and senescence in granulosa cells. Moreover, consistent with the results in the POF rat model, ADSCs significantly reduced apoptosis and senescence in CTX-treated granulosa cells. We also confirmed that ADSCs inhibited proliferation and reduced senescence in granulosa cells by Ki67 (Fig. 5E) and p21 (Fig. 5F) immunofluorescent staining. Taken together, these data demonstrated that ADSCs inhibited the proliferation of granulosa cells in primordial and primary follicles, thus preventing granulosa cells apoptosis and senescence due to the cytotoxic effect of CTX on proliferative cells.

### ADSC transplantation improves ovarian structure and function through the PI3K/Akt/mTOR pathway

**Fig. 6:**
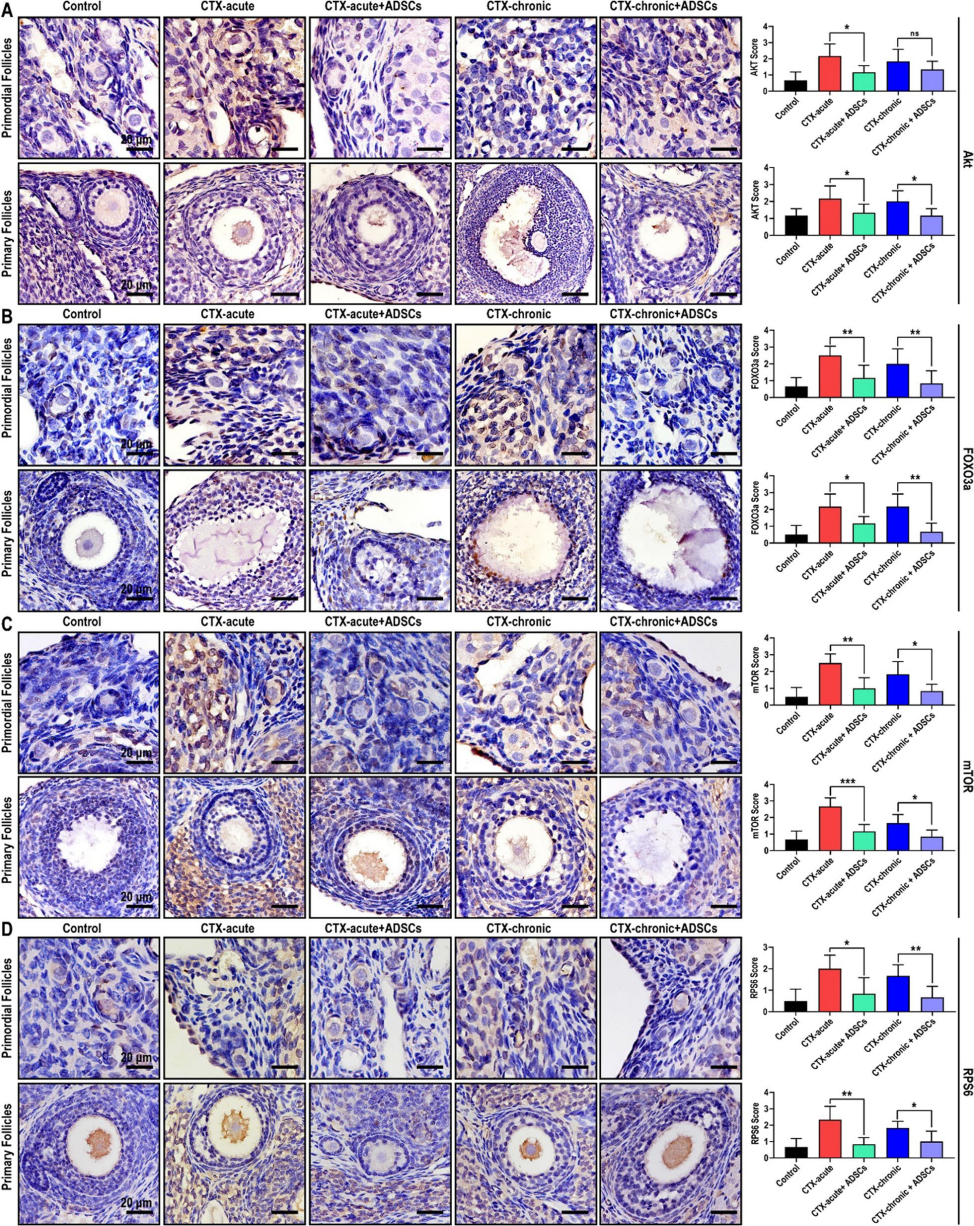
ADSC transplantation improves ovarian structure and function through the PI3K/Akt/mTOR pathway. The expression of Akt (**A**), FOXO3a (**B**), mTOR (**C**), and RPS6 (**D**) were significantly increased in rats with POF and decreased in rats treated with ADSCs

**Fig. 7:**
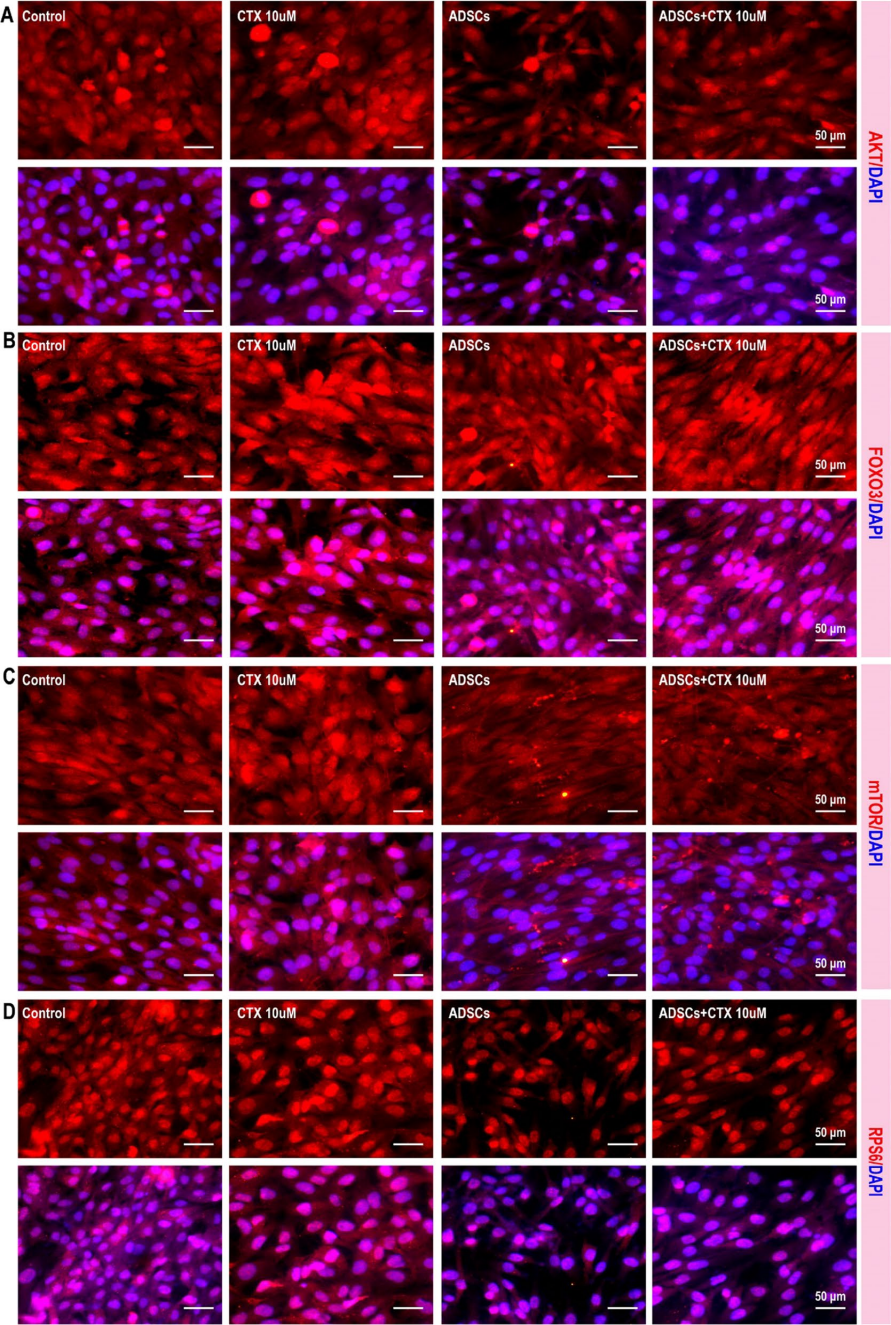
ADSC transplantation improves ovarian structure and function through the PI3K/Akt/mTOR pathway. The expression of Akt (**A**), FOXO3a (**B**), mTOR (**C**), and RPS6 (**D**) were significantly increased in rats with POF and decreased in rats treated with ADSCs

The improvement in ovarian function mediated by ADSCs might involve the inhibition of granulosa cell proliferation and apoptosis, but the underlying mechanism remains unknown. Notably, upregulation of the Akt/mTOR axis was involved in excessive activation-induced dysfunction and exhaustion of follicles. Therefore, we verified the expression of Akt/mTOR axis-related proteins in ovarian tissues of rats with POF, as well as rats with POF that were treated with ADSCs. As shown in Fig. 6, compared to control group rats, acute and chronic POF rats expressed significantly higher levels of Akt (Fig. 6A), FOXO3a (Fig. 6B), mTOR (Fig. 6C) and RPS6 (Fig. 6D). In our in vitro model, 10 µM CTX-induced senescence in granulosa cells, mimicking the dysfunction of ovarian hormone secretion. Consistent with the in vivo results, the expression of Akt (Fig. 7A), FOXO3a (Fig. 7B), mTOR (Fig. 7C) and RPS6 (Fig. 7D) was dramatically increased in senescent of granulosa cells. These findings indicate a close correlation existed between the senescence of granulosa cells and the upregulation of the Akt/mTOR axis. Then, we next examine whether ADSCs could regulate the AKT/mTOR axis. Our results suggested that after ADSC transplantation, the expression of Akt (Fig. 6A), FOXO3a (Fig. 6B), mTOR (Fig. 6C) and RPS6 (Fig. 6D) in acute and chronic rat granulosa cells was significantly inhibited. Furthermore, granulosa cells exhibited dramatically decreased expression of Akt/mTOR axis after being co-cultured with ADSCs (Fig. 7).

Overall, these data highlight that the Akt/mTOR axis plays a critical role in CTX-induced dysfunction in ovarian structure and function and demonstrate the close correlation of the beneficial effect of ADSCs in treating of POF and inhibition of the Akt/mTOR axis.

## Discussion

A substantial number of conventional therapeutic drugs, such as immunosuppressants and chemotherapeutic drugs are known to disrupt ovarian structure and hormone secretion [7, 42]. Cyclophosphamide is an alkylated drug that is widely used as an antitumor [43] and immunosuppressant drugs [44] and has the highest risk of POF. Therefore, many patients with cancer or autoimmune disease usually develop POF due to iatrogenic reasons. It is well-known that chemotherapy can induce cellular senescence [45] and apoptosis [46, 47], and proliferative ovarian cells are more susceptible to chemotherapeutic drugs-induced damage than other quiescent cells [48]. Previous studies suggested that chemotherapy-induced cellular senescence and apoptosis in follicles and stromal cells, lead to decreased ovary weight, disrupted ovarian structure and atrophy, and alterations in hormone secretion and fertility [49]. Cytotoxicity and dosage of chemotherapy drugs are the main determinants of the extent of ovarian damage induced by iatrogenic factors. Thus, in this study, we established two CTX-induced POF rat models: the acute POF model, which was administered a single high-dose injection of CTX, and the chronic model, which was administered multiple low-dose injections of CTX. The acute POF model mimics the monthly high-dose CTX treatment in clinic. The chronic POF model could provide better understanding of ovarian damage induced by low-dose injection of CTX and provide a new idea for reducing CTX cytotoxicity in clinic. We concluded that high-dose and low-dose CTX-induced POF rats exhibited primordial follicles depletions, and we further hypothesized that primordial follicles were activated by CTX and differentiated into primary and mature follicles. Moreover, we found that the body weight was decreased significantly and the estrous cycle disrupted within 2 weeks treated with CTX, thus providing clues for identifying the most suitable time for clinical treatment of POF.

POF is one of the main chemotherapy-related adverse effects in female patients with cancer. With the development of chemotherapy, the survival rate was increased and accompanied by increased incidence of POF [50]. Chemotherapy had deleterious effect on the function of hormone secretion and fertility of ovary [51]. Hormone replacement therapy is the commonly used treatment to only improve the symptoms induced by the deletion of hormone secretion in POF patients in the short term [52]. Therefore, there is a lack of safe and effective treatments to recover the damaged ovarian structure and secretion function of ovary currently. HRT lacks the long-term efficacy of improving hormone secretion rarely without adverse effects in patients with POF, and has little promotion of fertility preservation of patients [53]. In addition, lots of published studies also have demonstrated that long-term HRT increases the risk of breast cancer [54, 55]. However, ADSCs serve as a promising therapeutical option for patients with POF potentially recovery the damaged structure and secretion of ovary without increasing risk of cancer [56]. Sun et al. showed that ADSCs significantly increased the number of ovarian follicles and regulated the ovulation cycle in CTX-treated mice [57]. Huang et al. confirmed that exosomes secreted by ADSCs could repair the damaged structure and endocrine function of the ovary, and improve the microenvironment of ovarian tissue by upregulating the protein expression of Smad [58]. Ding et al. showed that HGF and bFGF were secreted by ADSCs and inhibited the levels of ROS by upregulating FoxO1 expression, thereby reducing ROS-induced damage to ovarian structure and function [59]. More importantly, ADSCs also displayed significant effect to recovery the fertility in rat model of POF. Su et al. confirmed that ADSC transplantation on collagen scaffolds could improve the fertility of rats after tripterygium glycosides (TG) induced ovarian injury. Their experimental results confirmed that the pregnancy rates in the ADSC and collagen/ADSC groups were significantly higher than those in the PBS group [60].

Our results also showed that body weight, the estrous cycle, and hormone secretion levels in rats quickly recovered to normal level after transplanted with ADSCs. Further analysis by HE staining showed that after transplanted with ADSCs, the loss of different types of ovarian follicles in rats was significant attenuated. Although the ovarian structure and function improved almost to normal level, it was also existed significant gap to healthy rats, indicating the protective effect of ADSCs on the structure and function of ovarian tissue. Considering the overactivation of primordial follicles in rats induced by CTX, we examined the proliferation and apoptosis in rats ovarian tissue after transplantation of ADSCs. The results showed that ADSCs significantly inhibited the proliferation of primordial follicles in rats, and significantly inhibited apoptosis in follicles, including primordial follicles, to retain more follicles in rat ovarian tissue, thus reducing the CTX-induced damage to rat oocyte tissue. Furthermore, ADSCs can also secrete VEGF, HGF, IGF and other cytokines, to promote the repair and angiogenesis of the injured site, improve the microenvironment of the injured site, and provide the basis for the incomplete treatment of POF.

Research on the molecular mechanism of POF is limited, and the associated signaling pathways need to be further studied. Since the granulosa cell growth and oocyte maturation of ovarian follicles directly affects the fertility and hormone secretion, any link in granulosa cell and oocyte that is affected by CTX will lead to ovarian disorders [61]. Research on the molecular mechanism of POF is limited, and the associated signaling pathways deserved further study. Therefore, it is critical to examine the pathogenesis of POF from the perspective of development. The etiology of POF involves genetic factors, immune system disorders, iatrogenic factors, mental stress and environmental toxicity [51], and understanding the mechanism of signaling pathways involved in the pathogenesis of POF has become a research hotspot and breakthrough point in recent years. The PI3K/Akt/mTOR signaling pathway plays a key role in the development of ovarian tissue. There is a complete PI3K/Akt/mTOR signaling pathway in oocytes and granulosa cells of ovarian follicles, which regulates the growth, development, maturation, and periodic ovulation of follicles [62]. Our data displayed that ADSCs displayed antiapoptotic and proliferation inhibition effects during treatment of POF. Mechanistically, ADSCs inhibit the intracellular PI3K/Akt/mTOR signaling pathway in ovarian tissue. Akt effectively activated primordial follicles in ovarian tissue, indicated that the Akt signaling pathway plays an important role in maintaining the balance between resting and activated primordial follicles. The PI3K/Akt/mTOR signaling pathway is regulated by ADSCs, regulates the growth of oocytes, and the survival and development of primordial follicles, inhibits apoptosis in follicular cells, plays a key role in maintaining the normal structure and function of ovarian tissue, and has a broad prospect for the prevention and treatment of POF.

## Conclusion

In summary, our work serves as the basis for the injection of ADSCs to facilitate the restoration of premature ovarian failure by suppressing apoptosis and senescence in granulosa cells and promoting hormone secretion. Our findings demonstrate that ADSC-based therapy might be a promising alternative for patients with POF especially for those who suffer CTX-induced acute POF and provide evidence for ADSC transplantation in future clinical trials.

## Data Availability

The datasets used and/or analyzed during the current study are available from the corresponding author.

## Abbreviations

POF: Premature ovarian failure
CTX: Cyclophosphamide
ADSC: Adipose-derived stem cell
AMH: Anti-Müllerian hormone
E2: Estradiol
FSH: Follicle-stimulating hormone
VEGF: Vascular endothelial growth factor
IGF-1: Insulin-like growth factor-1
HGF: Hepatocyte growth factor
SD rats: Sprague Dawley rats
DMEM: Dulbecco’s modified Eagle’s medium
